# Quantitative Analysis of Solar Photovoltaic Panel Performance with Size-Varied Dust Pollutants Deposition Using Different Machine Learning Approaches

**DOI:** 10.3390/molecules27227853

**Published:** 2022-11-14

**Authors:** Abhishek Kumar Tripathi, Mangalpady Aruna, Elumalai Perumal Venkatesan, Mohamed Abbas, Asif Afzal, Saboor Shaik, Emanoil Linul

**Affiliations:** 1 Department of Mining Engineering, Aditya Engineering College, Surampalem 533437, India; 2 Department of Mining Engineering, National Institute of Technology Karnataka, Mangaluru 575025, India; 3 Department of Mechanical Engineering, Aditya Engineering College, Surampalem 533437, India; 4 Electrical Engineering Department, College of Engineering, King Khalid University, Abha 61421, Saudi Arabia; 5 Electronics and Communications Department, College of Engineering, Delta University for Science and Technology, Gamasa 35712, Egypt; 6 Department of Mechanical Engineering, P. A. College of Engineering (Affiliated to Visvesvaraya Technological University), Mangaluru 574153, India; 7 University Centre for Research & Development, Department of Mechanical Engineering, Chandigarh University, Gharuan, Mohali 140413, India; 8 School of Mechanical Engineering, Vellore Institute of Technology, Vellore 632014, India; 9 Department of Mechanics and Strength of Materials, Politehnica University Timisoara, 300222 Timisoara, Romania

**Keywords:** PV panel, dust size, output power, machine learning, support vector machine regression, Gaussian regression

## Abstract

In this paper, the impact of dust deposition on solar photovoltaic (PV) panels was examined, using experimental and machine learning (ML) approaches for different sizes of dust pollutants. The experimental investigation was performed using five different sizes of dust pollutants with a deposition density of 33.48 g/m^2^ on the panel surface. It has been noted that the zero-resistance current of the PV panel is reduced by up to 49.01% due to the presence of small-size particles and 15.68% for large-size (ranging from 600 µ to 850 µ). In addition, a significant reduction of nearly 40% in sunlight penetration into the PV panel surface was observed due to the deposition of a smaller size of dust pollutants compared to the larger size. Subsequently, different ML regression models, namely support vector machine (SVMR), multiple linear (MLR) and Gaussian (GR), were considered and compared to predict the output power of solar PV panels under the varied size of dust deposition. The outcomes of the ML approach showed that the SVMR algorithms provide optimal performance with MAE, MSE and R^2^ values of 0.1589, 0.0328 and 0.9919, respectively; while GR had the worst performance. The predicted output power values are in good agreement with the experimental values, showing that the proposed ML approaches are suitable for predicting the output power in any harsh and dusty environment.

## 1. Introduction

Due to the surrounding environmental parameters, solar photovoltaic (PV) panels experience a significant variation in their performance whenever they operate in an open atmosphere [1]. Generally, the degradation of the PV panel performance depends on its internal design as well as external environmental parameters (where it is exposed). Among the environmental parameters, foreign particles, such as dust, largely contribute to the performance of the PV panel, while the other external parameters, such as atmospheric temperature, wind speed and humidity, help the foreign particles to spread and enter into the panel surface.

The deposition of dust over the panel acts as a barrier, which attenuates sunlight reaching the surface of the panel [2]. Due to this fact, the efficiency of PV panels is significantly reduced. The deposition of dust on the surface of the panel provides partial or complete shading of the PV panel [3,4]. The reduction of sunlight reaching the panel surface depends on the type, density and size of the deposited dust pollutants [5,6].

A study by Shobokshy and Hussein admitted that the decrease in the conversion efficiency of the PV panel was 10%, 16% and 20%, respectively, due to the accumulation of 12.5 g/m^2^, 25 g/m^2^ and 37.5 g/m^2^ of dust density [7]. In another study, the reduction of 92.11% in power generation capacity and 89% reduction in output efficiency of the panel due to dust deposition on its surface was reported [8]. The performance of the PV panel is directly dependent on the sunlight [9] and its yield current shows the linear relationship with the surface sunlight [10,11], while the output voltage shows the logarithmic relationship [12]. The reduction in the efficiency of the PV panel follows a linear relationship with the amount of dust deposited on its surface [13]. Elminir et al. [14] obtained a 40.02% reduction in glass transmittance due to the admission of a dust density of 11.34 g/m^2^.In a similar type of work, the spectral and glass transmittance of the panel showed a decrease of 35% and 20%, due to the deposition of 5 g/m^2^ [15]. Further, the study conducted by Kaldellis and Kapsali [16] confirmed that the panel yield power cuts by 19%, 10% and 6%, respectively, due to red soil, limestone and ash particle deposition. Similarly, in [17], panel effectiveness was examined under city dust pollution and it was observed that regular and white cement result in greater reduction in output power compared to sand and gypsum. A study on the effect of dust accumulation and different cleaning methods showed that a salty cohesive layer was formed due to the interaction of dust and moisture on the panel surface [18]. This layer requires a large amount of cleaning power to clean the panel surface. In addition, the size, shape and color of dust pollutants also affect the efficiency of PV panels by reducing their light transmission capacity. Fountoukis et al. [19] indicates that size is also an important factor determining panel degradation in a dusty climate, and it is observed that particles of different sizes give different performance values at the same deposition density. This encourages more studies to understand the dimensional effect of dust deposition on PV panel operating efficiency. In addition, the small dust particle size covers a larger area than the large particle size, which means that the finer dust size provides more traffic in the path of sunlight trying to penetrate the panel surface.

Recent works highlighting the dust deposition and discoloration of PV panel performance are presented in Table 1.

Most previous research has focused on dust density and dust type when examining panel performance, but the study on dust size is very limited, indicating that more studies are needed in this area. Thus, the main purpose of this paper is to understand the influence of dust size on the performance of PV panels, which would further help in locating the site for installing solar panels. In this regard, the machine learning (ML) approach is used to predict the output power due to the allowance of varying size of dust pollutants on the panel surface. Introducing ML into data prediction provides a high real-time prediction value that ensures better decision and intelligent action without any human intervention. The main advantage of ML is that it works on both structured and unstructured data during the preparation of the prediction model [27,28]. Different ML approaches have been used to predict the output power of solar PV panels, but in all previous studies, the output power prediction was made based on environmental parameters (solar irradiance, ambient temperature, and humidity). There has not been a considerable study reported by researchers that could predict the power of the panel based on the amount and size of dust pollutants deposited on its surface. Thus, this drawback of previous studies motivates the authors to perform the present research work. Furthermore, from previous studies, it is evident that the sunlight on the panel surface is directly proportional to its performance. Therefore, an attempt was made to establish the relationship between solar irradiance and the output current of the panel. With the help of this developed relationship, the performance analysis of the PV panel under the deposition of various sizes of dust pollutants was carried out; the main purpose of this work is to justify the experimental outcomes. This justification of the experimental results is the main novelty of the present research work, that clearly explains how the amount of solar irradiance (those penetrating inside the panel glass) is reduced for the small size of dirt on the panel surface. This would benefit solar power producers and consumers in designing and efficiently maintaining solar PV panels in harsh and dusty environments. All these studies were carried out using an experimental set-up, which is performed in the laboratory environment. 

This paper includes the sections as follows: Section 2 presents the research methodology and experimental set-up that is adopted for the present study. Section 3 presents the ML approach to predict the output power of the PV panel under the deposition of varying sizes of dust pollutants. Section 4 establishes the relationship between solar irradiance and output current, which is followed by Section 5, in which the performance analysis of the PV panel was made, regarding deposition of various sizes of dust pollutants. Section 6 of the paper is where the conclusions of the entire research paper are presented.

## 2. Research Method and Experimental Set-Ups

**Figure 1:**
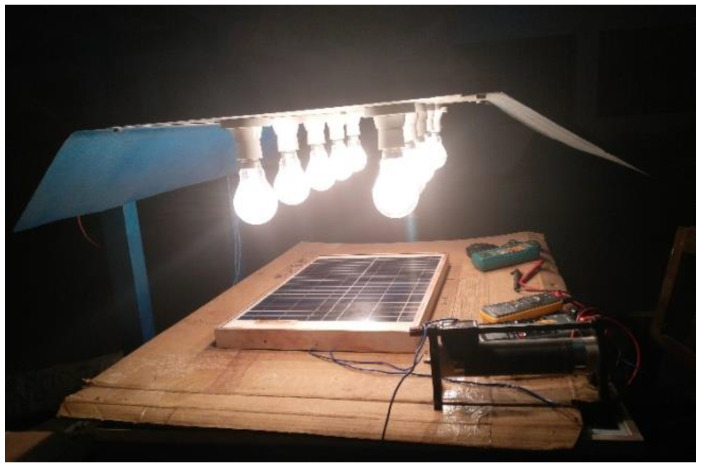
Experimental set-up.

The main aim of the present research work is to understand the effect of dust pollutant size on the performance of PV panels. To achieve this, the research work is divided into three different objectives. The first objective is to predict the output power of solar PV panels using different ML approaches under deposition of different sizes of dust pollutants. The second objective is to establish the relationship between the short-circuit current of the PV panel and solar irradiance. The third objective is to analyze the performance of the PV panel due to the deposition of different sizes of dust pollutants. To achieve all three objectives, it is necessary to carry out a laboratory study that provides a data set on which to perform the analytical work. Therefore, a test setup was designed to inspect the behavior of the PV panel when covered with dust particles. Figure 1 shows the photographic image of the experimental setup, which includes a 1 m high support with a level edge on it to hold the panel horizontally. A 20 W polycrystalline solar PV panel was mounted on the flat frame, consisting of two parallel strings with 18 cells in each string. A set of ten incandescent bulbs (200 W—5 nos. and 100 W—5 nos.) was used as the solar irradiance source (i.e., in the range 449 W/m^2^ to 920 W/m^2^). This solar irradiance range was used due to the experimental limitations. In this study, the lower radiation range was kept at449 W/m^2^, because below this range a very low short-circuit current could be observed, which does not support the panel characteristics graph (i.e., I-V and P-V curves). The solar power meter was used to measure the intensity of radiation falling on the PV panel surface in W/m^2^ [29].

The experimental laboratory study was carried out under controlled environmental conditions, i.e. the ambient temperature, humidity level, wind speed, etc., were kept constant. Further, humidity and ambient temperature were maintained at 65.40% and 31.2 °C, respectively, during the experiment. To understand the impact of dust pollutant size on the operating behavior of solar PV panels, dust samples were collected from an iron ore mine, and to separate dust particles of different sizes, sieve analysis was performed (gradation test). In the gradation test, different mesh sizes were used, ranging from 850 to 75 µ, and, based on the sieve size, the pollutants were designated from larger to smaller. Table 2 indicates the size of dust particles used in this study.

A voltmeter and an ammeter were used to measure the yield value of the PV panel in terms of voltage and current, respectively. The voltmeter used in this study has a maximum limit of 600 V DC with measurement accuracy of 0.5% and the ammeter has a maximum limit of 10 amps with a measurement accuracy of 2%. Initially, the output electrical responses of PV panels (such as voltage, current and power) were measured for clean surface panel under a laboratory environment, which was generated using the artificial solar simulator, and was maintained at a constant throughout the study. For the measurement of each electrical response, firstly, a reading of the solar power meter was taken, which gave the value of solar irradiance (it was maintained at 750 W/m^2^ using artificial light simulators). Thereafter, the output load of the panel (linear rheostat) was moved from the open-circuit position to the short-circuit position and readings of current and voltage were noted. The value of the measured output voltage of the PV panel at the open-circuit position of load gives the value of its open-circuit voltage and, in a similar manner, the value of the output current at the short-circuit position gives its short-circuit current value. In plotting the panel characters, these values are helpful. Further, the load was raised by turning the rheostat knob, from short-circuit to open-circuit position, prior to taking each reading of output current and voltage of the panel. Every reading was recorded one-at-a-time, waiting until the readings’ fluctuations stopped. The measurements were carried for the final step of the rheostat, until the open-circuit voltage reading was achieved. At this point, the value of the output voltage was maximum and current was minimum (near zero). It was observed that voltage readings increased while current readings decreased. The panels were then polluted by the deposition of dust particles and the same procedure was undertaken. Here, the electrical responses of the PV panel were recorded by spreading the dust pollutants of five different sizes (i.e., T1, T2 T3, T4 and T5) on the panel surface, separately, in a mass of 5 g (i.e., 33.58 g/m^2^ dust density) and the same procedure was undertaken. The dust pollutants were spread over the panel surface intensely by manual operation so that the same dust density on the panel surface could be maintained for all sizes of dust pollutants and this would help in investigating the solo effect of dust particle sizes on the PV panel performance.

The obtained readings from the laboratory experiments were used as a data set for predicting the output power of the panel using a machine learning (ML) algorithm. There are some studies that incorporated the use of machine learning algorithms to predict the output of solar photovoltaic panels [30,31]. In previous studies, it was reported that the prediction of PV panel output using ML was only focused on the training and testing of one machine learning algorithm, while other algorithms that could be tested for output power prediction were not examined. Therefore, in this paper an attempt has been made to predict the output power of solar PV panel using different machine learning algorithms. The selection of ML algorithms was based on the structure and complexity of the experimental data. Predictions were made of the output power of solar PV panels for different sizes of dust pollutants deposition on the panel surface (which ranged from T1 to T5 as indicated in Table 2). Thus, the main input variable for the prediction of output power was dust size, and other parameters such as solar irradiance, dust density, and panel temperature were considered constant. Different ML approaches such as support vector machine regression (SVMR), Gaussian process regression (GPR) and multiple linear regression (MLR) were used for predicting and determining suitable algorithms. Thereafter, the relation between short-circuit current and solar irradiance was established using a linear regression approach so that the reduction in solar irradiance level could be examined under deposition of varying dust pollutant sizes.

## 3. Machine Learning Approach for Prediction of Output Power

In this section, the output power of the solar PV panel is predicted using a different ML approach. The output power was predicted for a dust deposition density of 33.48 g/m^2^ with different pollutant sizes ranging from T1 to T5 (as indicated in Table 1) under constant solar irradiance of 750 W/m^2^.

**Figure 2:**
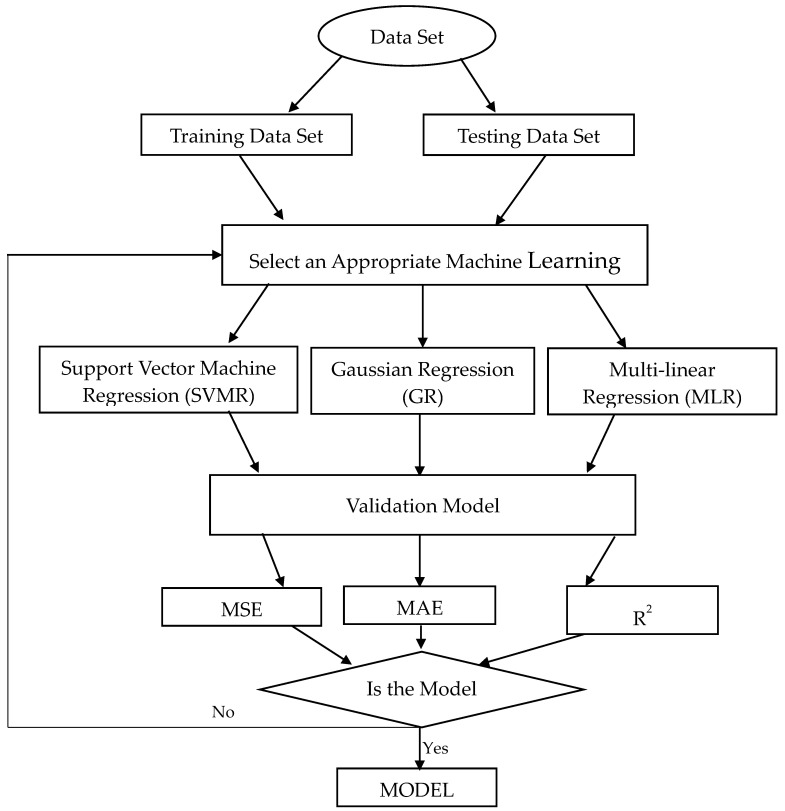
Machine learning workflow for output power prediction.

The ML algorithm is selected in such a way that it predicts the output power of the PV panel under the deposition of larger and smaller sized dust mass in a more appropriate manner. ML is a subset of artificial intelligence (AI) that explores construction and analysis algorithms that make predictions on data. Here, support vector machine regression (SVMR), Gaussian regression (GR) and multi-linear regression (MLR) machine learning algorithms were used to predict the output power under the deposition of different sizes of dust pollutants. The input variables such as solar irradiance, dust deposition density, dust particle size, output current and output voltage were collected from the laboratory experiment. The dataset used was pre-processed before being passed to the ML algorithms. The workflow of the proposed regression model is presented in Figure 2.

### 3.1. Support Vector Machine Regression (SVMR)

Support vector regression works on the principle of support vector machines (SVMs), which can be applied for both classification and regression analysis. It is used to predict discrete values and find the best fit line for the model. In this, the best fit line is the hyperplane that has the maximum number of points that can be identified through the kernel space. The kernel spaces can be reproduced by support vector machines regression (SVMR) with a loss function, which is called the insensitive loss function (ILF) and is denoted by ε. The empirical error in the SVMR model is measured by the ILF, i.e., ε, which is more robust and sparser. The SVMR model is more flexible, in that it allows us to use linear and non-linear relationships between variables, which makes it more robust. The SVR helps to achieve generalized performance of the model by minimizing training and generalized error [32]. SVR identifies data non-linearity and provides an expert prediction model. Let us consider the m-dimensional training values containing k data points {Y1, Y2, …, Ym} and the target model data {a_1_, a_2,_ …, a_m_} then the evaluation of a, which is represented by t, is stated in Equation (1) [33,34].

(1) t=ω φ(Y)

where *ω* and *φ* denote the weight vector and the nonlinear mapping in the kernel space. This nonlinear mapping in kernel space can be improved by using Equations (2) and (3). These equations optimize the complex behavior of the input data and differ from each other based on the slack variables and the cost functions, *V_i_* and *V_i_i*, which are defined in Equation (4).

(2) $$a_{i}-\omega T\varphi \left(X_{i}\right)\leq \varepsilon +V_{i},\;i=1,\;2,\;\ldots ,\;M$$

(3) $$\omega \;T\;\varphi (X_{i})-a_{i}\leq \varepsilon +V_{i},\;i=1,2,\ldots \ldots .,M$$

(4) $$V_{i},V_{i}i\geq 0,\;i=1,2,\ldots \ldots .,M\;$$

The value of the slack variables and the cost functions, as mentioned in the above equations, must be minimized in nature so that the accurate optimization of SVR model can be achieved, and the constrained optimization is given by Equation (5).

(5) $$\min A(\overset{r}{\underset{i=1}{\sum }}\left(V_{i\;}+{\bar{V}}_{i}\right))+\frac{1}{2}\vec{\omega }\bar{\omega }$$

where A is called regularization constant.

### 3.2. Gaussian Regression (GR)

In ML models, the GR model is among those few models that can solve any complex system analytically by a non-parametric Bayesian approach [35]. The Bayesian non-parametric method helps to select the model at an appropriate level of complexity that consists of random variables and provides the Gaussian distribution profile for all input–output data sets. The GR process provides are liable estimate of its own uncertainty and also provides more flexibility to the prediction model in terms of interpolation. For the mean function m (i) and the kernel function p (i, i’), the GR can be defined by Equation (6), where m (i) denotes the central leaning of the input Gaussian function F (i), which is related to test output function O (i) as mentioned in Equation (7). The input and output function of the GR is related to the noise term ε.

(6) $$F\left(i\right)=\mathrm{GP}\left(m\left(i\right),\;p\left(i,\;i^{\prime }\right)\right)$$

(7) O(i)=F(i)+ε

here, ε denotes the independent noise term which is a function of variance V_m_ and is defined by Equations (8) to (11).

(8) $$\varepsilon =D\left(0,v_{m}^{2}\;\right)$$

(9) $$H\left(o|f\right)=D\left({y|v}_{m}^{2}\;J\;\right)$$

here

(10) $$O={\left[O_{1},O_{2},O_{3}\ldots \ldots \ldots .O_{n}\right]}^{T\;}$$

(11) $$f={\left[f\left(i_{1}\right),f\left(i_{2}\right),f\left(i_{3}\right)\ldots \ldots \ldots .f\left(i_{n}\right)\right]}^{T}$$

### 3.3. Multiple Linear Regression (MLR)

To establish the linear relationship between a single dependent variable and more than one independent variable, MLR might be the best choice. The MLR model works better for more complex relationships that require more specific calculation by properly considering the dataset. It helps in predicting the behavior of response variables relative to the movement of predictor variables. The performance of MLR can be examined by root mean squared error (RMSE), mean absolute error (MAE) and coefficient of determination (R^2^), and these values can be analyzed by Equations (12)–(14).

(12) $$\mathrm{RMSE}=\sqrt{\overset{N}{\underset{i=1}{\sum }}\frac{{\left(p_{i}-{\;p^{\prime }}_{i}\right)}^{2}}{N}}$$

(13) $$\mathrm{MAE}=\overset{N}{\underset{i=1}{\sum }}\frac{\left|p_{i}-{p^{\prime }}_{i}\right|}{N}$$

(14) $$R^{2}=1-\frac{\sum_{i=1}^{N}{\left(p\prime_{i}-p_{i}\right)}^{2}}{\sum_{i=1}^{N}{\left(p\prime_{i}-p\prime_{ave}\right)}^{2}}$$

here, N is the total number of data points. pi, p’_i_ and p’_ave_ represent the predicated value, target value and the average target value, respectively.

## 4. Prediction of Output Power Due to Size-Varied Dust Pollutants Deposition

The outcome of different ML algorithms applied to predict the power of the PV panel under the accumulation of varying dust sizes is presented in this subsection. To analyze the experimental data, the entire data set is divided into training and testing datasets. The training and testing part consists of 75% and 25% of the data set. Here, the four-fold cross-validation technique is applied on the dataset, where three-folds are used for training and one-fold is used for testing. The results of the four-fold cross validation technique for three different ML algorithms are shown in Table 3. The average value of the performance parameter gives the results of the ML algorithm for output power prediction.

**Figure 3:**
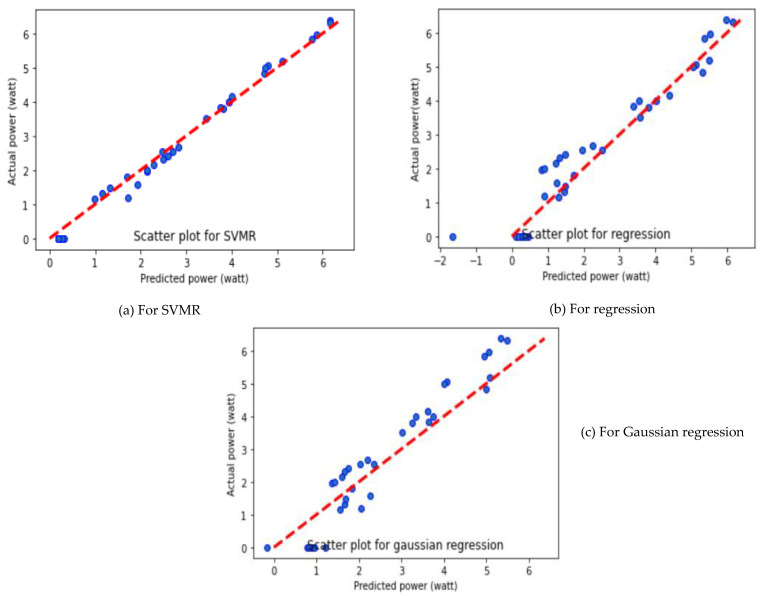
Prediction of output power of solar PV panel using different ML algorithms for Fold-1.

As depicted in Table 3, SVMR algorithms provide better performance with different average performance parameters such as MAE, MSE and R^2^ value of 0.1589, 0.0328 and 0.9919, respectively. It is further observed that the GR algorithm showed the worst performance, with MAE of 0.6125, MSE of 0.4778 and R^2^ of 0.8808. The GR displayed poor performance, due to the complex relationship between the yield value of the PV panel and the input variables. The output power prediction using different ML algorithms is presented in Figure 3.

**Figure 4:**
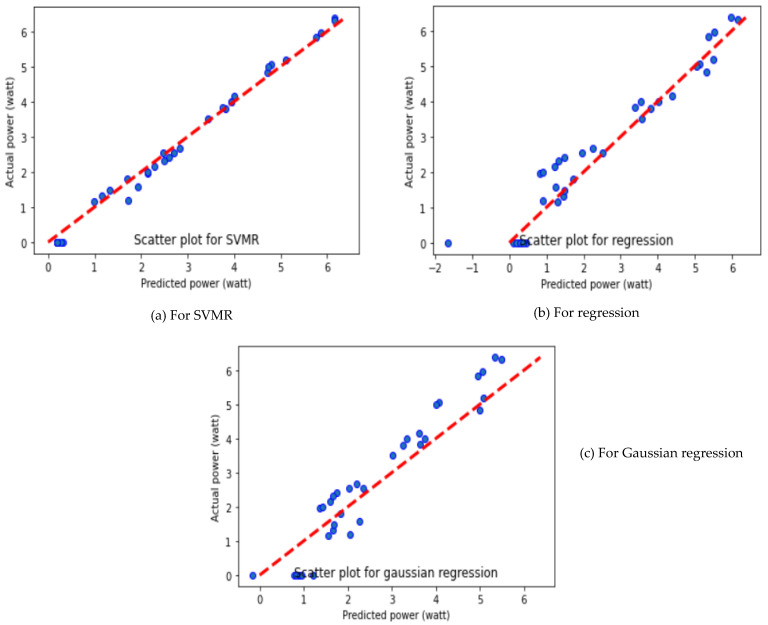
Prediction of output power of solar PV panel using different ML algorithms for Fold-2.

**Figure 5:**
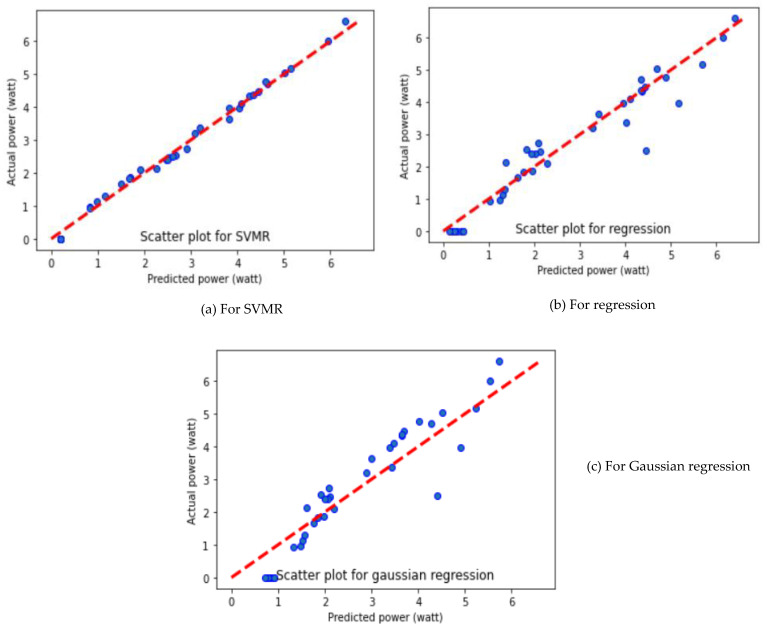
Prediction of output power of solar PV panel using different ML algorithms for Fold-3.

It can be seen that SVMR predicted well in comparison to MLR and GR algorithms. However, the SVMR and MLR prediction algorithms show the predicted value closer to the actual data set, while the GR algorithm showed a large deviation, offset from the centerline. The viability of the obtained results is demonstrated by comparing the predicted results with the experimental results. The relationship between the predicted output powers and the actual output power is plotted for three different folds and presented in Figure 3, Figure 4 and Figure 5. The reason for SVMR’s good performance is its ability to manage the best fit line within threshold values (also called the epsilon-insensitive tube). Moreover, the collected data sets were classified based on the different sizes of dust pollutants, and SVMR performed well in the classification data sets because of its ability to choose the decision boundary that maximizes the distance from the nearest data points of all the classes.

## 5. Establishing the Relationship between Solar Radiation and Short-Circuit Current

The yield response of the PV panel was recorded for 10 different values of solar irradiance, i.e., 449 W/m^2^, 516 W/m^2^, 593 W/m^2^, 608 W/m^2^, 661 W/m^2^, 719 W/m^2^, 740 W/m^2^, 788 W/m^2^, 878 W/m^2^ and 918 W/m^2^ under different output load conditions, and their values are given in Table 4.

**Figure 6:**
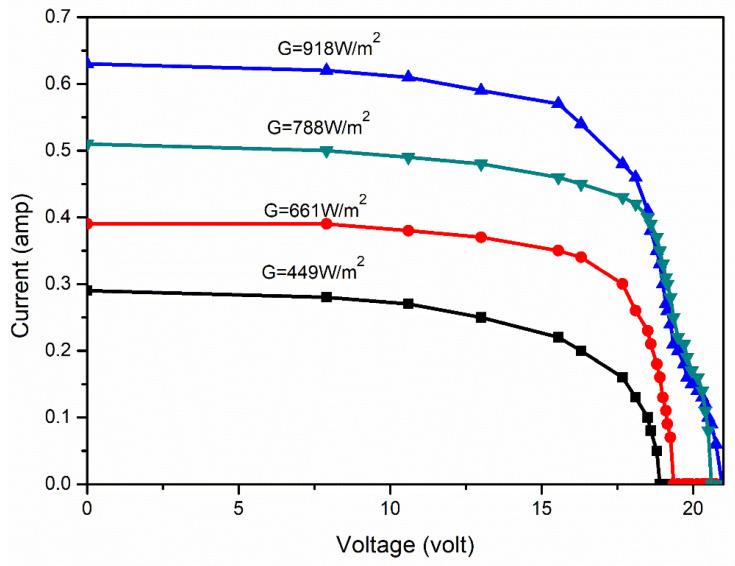
I-V curves of the solar PV panel for different solar irradiance.

**Figure 7:**
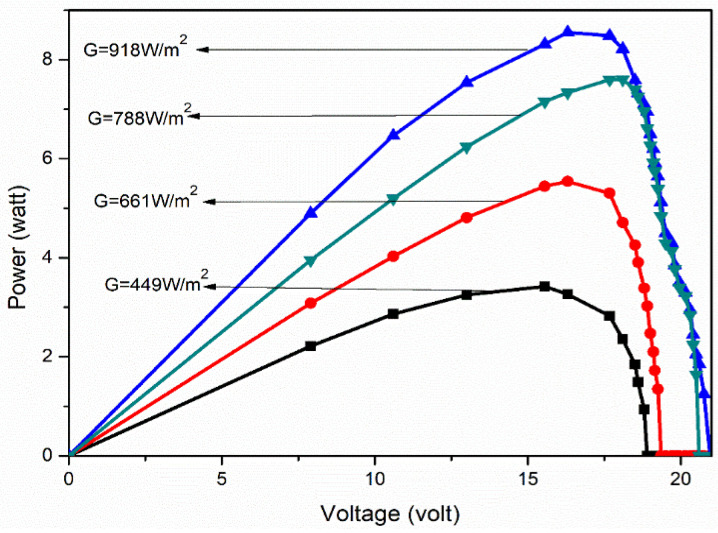
P-V curves of the solar PV panel for different solar irradiance.

The obtained yield values of the PV panel were used to plot the current–voltage curves for four different solar irradiance levels, i.e., 449 W/m^2^, 661 W/m^2^, 788 W/m^2^ and 918 W/m^2^, and these curves are shown in Figure 6 and Figure 7. The plotted curves demonstrate the working of PV panels under different levels of solar irradiance, which states that during the initial stage of the curves, the output current is almost constant, but suddenly drops to zero due to the semiconductor nature of the panel. Similarly, in Figure 5, the output power shows the linear relationship with the output voltage and then drops to zero, which is called the panel-voltage-under-no-load condition.

**Figure 8:**
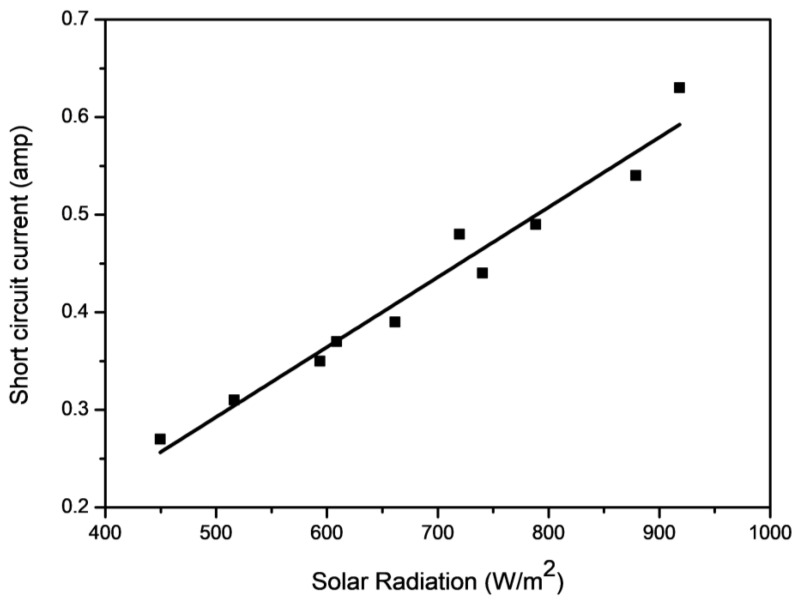
Output current with solar irradiance.

**Figure 9:**
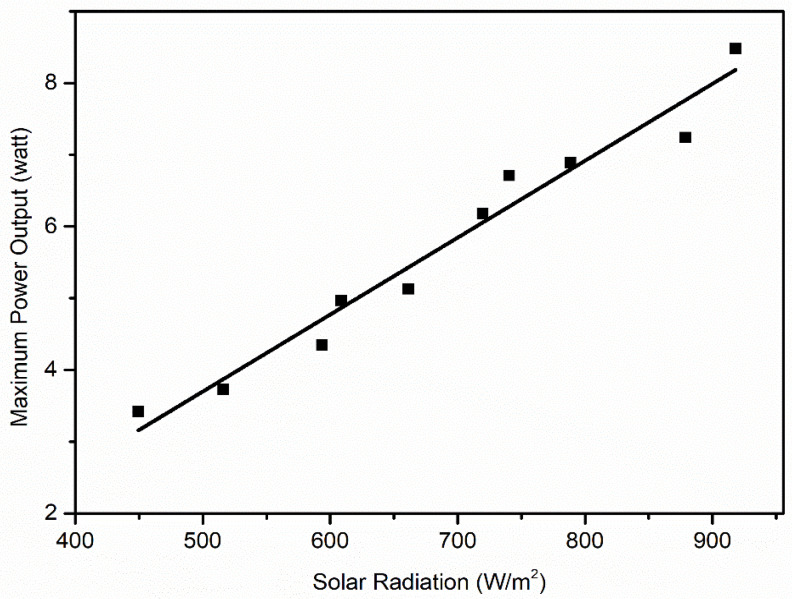
Output power with solar irradiance.

**Figure 10:**
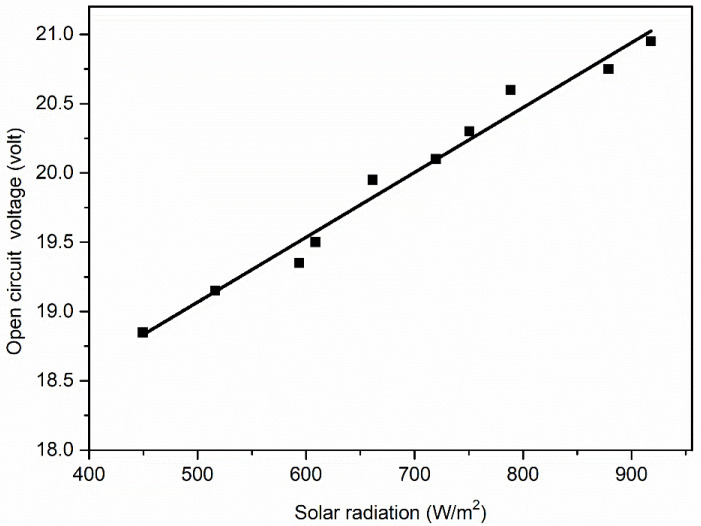
Output voltage with solar irradiance.

As can be seen from Figure 6 and Figure 7, the amount of solar irradiance affects the output power and current more predominantly than the voltage. Thus, it can be concluded that any change in the surface radiation of the PV panel significantly affects the output current and power. Generally, the admission of dust particles to the surface of the panel alters the surface radiation ability. Therefore, to understand the change of surface radiation, it is necessary to plot the relationship curve between the output current, power and voltage with solar irradiance (Figure 8, Figure 9 and Figure 10).

From Figure 8, Figure 9 and Figure 10, it can be seen that the short-circuit current (I_sc_)and maximum power output (P_max_) have a more linear relationship with solar irradiance(G) than open-circuit voltage (V_oc_). Therefore, the linear regression model is used to establish the relationship between I_sc_ and G, and the data used for this regression are shown in Table 5. This strategy helps in setting up the connection between the dependent and independent variables. The purpose of straight-line regression is to find the best fitting line that provides a strong fit to the measured data points, which can be seen in Figure 8. In Figure 8, all the observed data sets are closely fitted in a straight line, which is the most favorable condition for applying the linear regression method between G and I_sc_, and therefore applied.

The data values in Table 5 are analyzed using the linear regression technique, and the process used in the linear regression analysis is shown in Equations (15)–(17).Here, the independent variable I_sc_ is denoted as short-circuit current, which is analyzed for the n number of data points and the solar irradiance G is denoted as the dependent variable. The parameters a_0_, a_1_ and a_2_ are known as regression constants. Based on the defined regression equations, the model developed for Isc prediction is given in Equation (18).

I_SC_ = a_0_ + a_1_ G (15)

∑I_SC_ = na_0_ + a_1_ ∑G (16)

∑I_SC_ × G = a_0_ ∑G + a_1_ ∑G^2^ (17)

∑ I_SC_ = −0.062 + 0.0007G (18)

The coefficient of determination can be calculated by knowing the value of the correlation coefficient. The coefficient of determination is nothing but the square value of the correlation coefficient. The formula used to calculate the coefficient correlation is given by Equation (19).

(19) $$R^{2}=\frac{n\sum G\sum I_{sc}-\sum G\sum I_{sc}}{\sqrt{(n\sum G^{2}-\left(\sum G{)}^{2}\right)}(n\sum I_{sc}{}^{2}-{\left(\sum I_{sc}\right)}^{2})}$$

where: I*_SC_* = short-circuit current of PV panel (amp), *G* = solar irradiance falling on the PV panel surface (W/m^2^), *n* = number of observations, and *R* = coefficient of correlation.

The performance of the developed regression model is shown in Table 6. From Table 6, it is noted that the obtained model has a very good performance with coefficient of determination and mean square error value of 0.9612 and 0.02306. These values describe strong statistical parameters and can be considered for future prediction [36].

**Figure 11:**
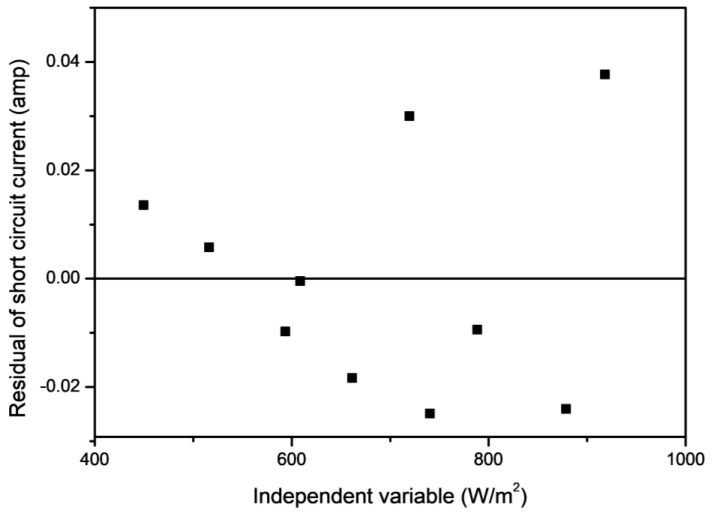
Residual plot for the developed mathematical relation.

To estimate the goodness of the developed mathematical model, the residual analysis method was used, and the data required in this analysis is presented in Table 7. A residual analysis method consists of the residual curve that is plotted between the residual of the developed model and the independent variable (see Figure 11). The residuals are nothing but the difference between the actual values and the estimated values (which are calculated by the developed mathematical model). The linear regression model can only be considered when the residuals are scattered in a random nature, otherwise non-linear regression should be preferred [37,38]. From Figure 11, it can be seen that the residuals are randomly dispersed, which supports the linearity of the developed model.

## 6. Analysis of PV Panel Performance under the Size-Varied Dust Pollutants Deposition

**Figure 12:**
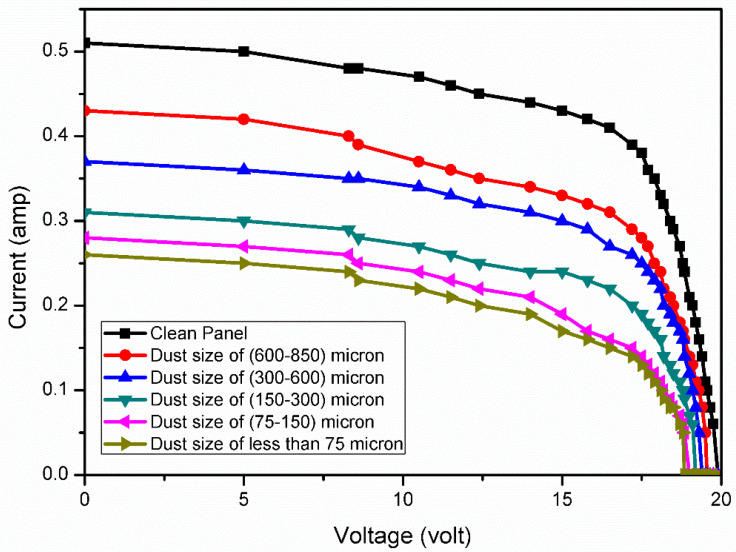
I-V curves of the solar PV panel for different dust size.

**Figure 13:**
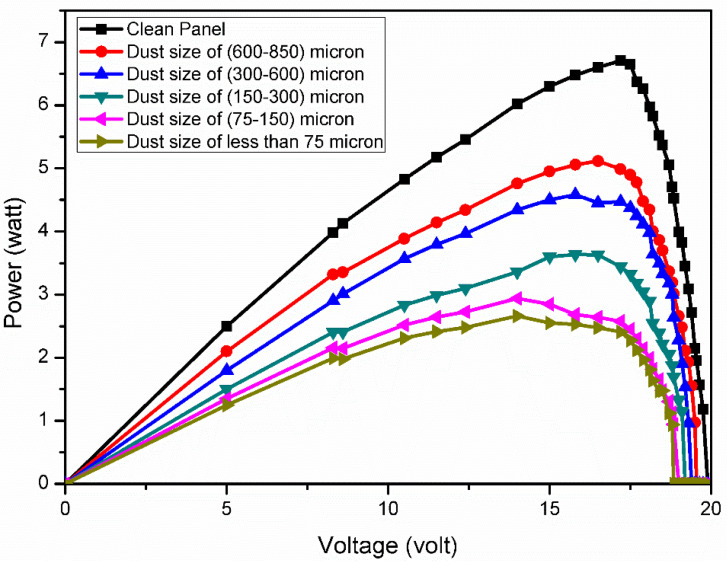
P-V curves of the solar PV panel for different dust size.

The output of the PV panel, such as current, voltage and power, were measured for five different sizes of dust particles (i.e., T1, T2, T3, T4 and T5) and its performance was compared with that of a clean PV panel (which is designated as T0), under the same set of conditions (i.e., same G). The electrical characteristics of PV panel, namely I-V and P-V characteristics, for all sizes of dust along with the clean panel were measured (under constant G of 750 W/m^2^) and plotted (Figure 12 and Figure 13).

From Figure 12, the percent reduction in Isc of the PV panel was calculated using Equation (20) for different sizes of dust particles, and shown in Table 8.

(20) $$RSCC=\frac{I_{\mathrm{sc},\mathrm{clean}}-I_{\mathrm{sc},\mathrm{dusty}}}{I_{\mathrm{sc},\mathrm{clean}}}\times 100\;\left(\%\right)$$

where, *RSCC* =reduction in short-circuit current (%), I_sc,clean_ = short-circuit current of clean PV panel, and I_sc,dusty_ = short-circuit current of dusty PV panel (amp).

**Figure 14:**
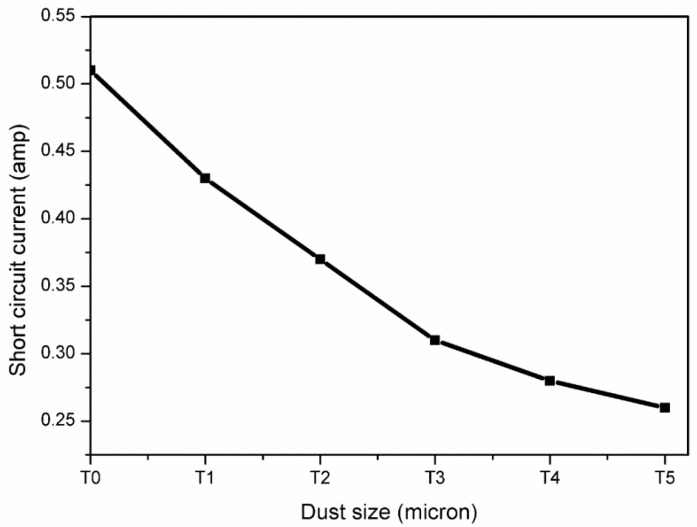
Short-circuit current relation with the deposition of different dust size.

The relationship of the output short-circuit current of the PV panel with the size of the deposited pollutant is shown in Figure 14.

The panel characteristics plotted revealed that the deposition of small size particles had a more serious impact on the output values of the panel than the large size. This is mainly due to the more adhesive nature of finer dust particles than coarser particles [39]. Thus, larger sized dust particles allow more sunlight to penetrate inside the panel glass than smaller sized particles (see Table 9) [40].

Therefore, the performance degradation of the PV panel is severe for smaller dust particles. A similar finding was observed by Adıgüzel et al [41] in their study. They compared the output power loss of the panel under artificial dust pollution of the panel surface with the six different sizes, and observed that the smaller size of the dust pollutants causes bigger reduction than the larger size.

With the help of Equation (18), the different values of solar irradiance were calculated for the different values of I_sc_ (which are mentioned in Table 8) and presented in Table 9. The calculated solar irradiance is called G_s_ and is compared to the reference value G_r_, so that the percentage reduction in the solar irradiance (*RSI*)due to the admission of different dust particles on the surface of the panel can be recorded.

(21) $$RSI=\frac{G_{r}-G_{s}}{G_{r}}\times 100\;\left(\%\right)$$

The value of G_r_ was calculated (as 800 W/m^2^) from Equation (21), considering the 0.48 amp value of I_sc_. Since the calculated value has 6.66% of the relative error with reference to actual recorded value, G_r_ is considered for analyzing the effect of dust size on solar irradiance values falling on the panel surface.

From Table 9, it can be seen that finer dust particles allow a shorter penetration path to sunlight (which reaches the panel surface) than coarser particles. The same finding was reported by [42,43], where the authors noted that the finer deposition of dust on the panel surface has a more significant effect on the solar irradiances penetrating its surface. It was observed that the solar irradiance showed a 40% reduction due to the deposition of smaller particles compared to the larger ones. This is mainly due to the larger area covered by smaller particles than larger ones under the same deposition density [44].

## 7. Conclusions

Solar-powered photovoltaic (PV) panels could be considered one of the main sources of sustainable electrical power generation. Generally, solar PV panels are exposed to an open environment where their performance is affected by various environmental parameters. Dust deposition on the panel surface is one of the frequently occurring environmental phenomena for the loss of output power of PV panels. The main purpose of this work is to understand the effect of different sizes of dust pollutants on the output power of the PV panel. In this work, the output power, due to the deposition of the different sizes of dust pollutants, was analyzed through experimental investigations and modeling of experimental data. Machine learning (ML) approaches such as support vector machine regression (SVMR), Gaussian process regression (GPR) and multiple linear regression (MLR) were used to predict the output power of the panel due to the accumulation of various sizes of iron ore dust pollutants. The outcomes of the ML approach showed that the SVMR algorithms provide the highest performance with MAE, MSE and R^2^ values of 0.1589, 0.0328 and 0.9919, respectively. This reveals that the developed ML model has high reliability and accuracy to predict the output power of solar PV panels. An output power prediction would benefit power producers and consumers in the efficient design and maintenance of solar PV panels in harsh, dusty environments.

Five different sizes of iron ore dust with a mass of 5 g were considered on a PV panel, with an area of 0.1489 m^2^, during the experimental investigation. A short-circuit current reduction of 15.68% was observed for deposition with large size dust particles (ranging from 600 µ to 850 µ), while for smaller dust sizes (i.e., less than 75 µ) the decrease was 49.01%. In addition, the performance of PV panels has shown that smaller size dust particles allow less solar irradiance on the panel surface than larger size particles, although the mass distribution is the same. This study demonstrated that under constant dust deposition mass there is a significant reduction of nearly 40% in solar irradiance as dust size varies from larger to smaller. This shows that smaller size dust particles affect the PV panel performance more significantly than larger size. The analysis of dust cleaning frequency, for PV panel surfaces, in relation to dust size, can be considered as future work, following this research article.

## Figures and Tables

**Table 1:** Study and outcomes of dust effect on PV panel performance.

Nature of Study	Work	Measure Parameter	Outcomes	References
Outdoor	Effect of dust discoloration on PV panel material	Glass Transmittance	15% degradation	[20]
Indoor	Effect of various types of dust deposition on mono and poly PV panel performance	Output power	Mono—12% degradation Poly—5% degradation For Ash deposition	[21]
Indoor	Type of dust impact on PV panel performance	Efficacy	Dust degrades the panel efficacy	[22]
Outdoor	Effect of desert dust on PV panels	Output power	98.13% reduction	[23]
Indoor	Investigation of PV efficacy under the deposition of different types of dust pollutants	Power	99.76% Performance reduction due to carbon dust	[24]
Outdoor	Proposed a model to encounter the impact of dust on grid-connected PV system	Inverter efficacy (IE) and performance efficacy (PE)	94% IE 73% PE	[25]
Indoor	Study the impact of coal dust	Output power	62.05% degradation	[26]

**Table 2:** Different sizes of dust particles and their designation.

Sl. No.	Particle Size Range	Symbolic Representation	Designated As
1	600 µ to 850 µ	T1	Larger size
2	300 µ to 600 µ	T2	Moderate larger size
3	150 µ to 300 µ	T3	Medium size
4	75 µ to 150 µ	T4	Moderate medium size
5	less than 75 µ	T5	Smaller size

**Table 3:** Results of ML algorithms for solar PV panel output power.

Algorithms	Performance Parameters	Fold-1	Fold-2	Fold-3	Average Value of Performance Parameters
SVMR	MAE	0.1708	0.1708	0.1351	0.1589
	MSE	0.0381	0.0381	0.0224	0.0328
	R^2^	0.9910	0.9910	0.9937	0.9919
MLR	MAE	0.3926	0.3926	0.3256	0.3702
	MSE	0.2833	0.2833	0.2377	0.2681
	R^2^	0.9334	0.9334	0.9334	0.9334
GR	MAE	0.6301	0.6301	0.5773	0.6125
	MSE	0.4906	0.4906	0.4523	0.4778
	R^2^	0.8847	0.8847	0.8732	0.8808

**Table 4:** Yield value of the panel under different solar irradiance.

Solar Irradiance (W/m^2^)	Maximum Power Output (W)	Short-Circuit Current (A)	Open-Circuit Voltage (V)
449.58	3.42	0.27	18.85
516.22	3.73	0.31	19.15
593.70	4.35	0.35	19.35
608.63	4.97	0.37	19.50
661.49	5.13	0.39	19.95
719.60	6.18	0.44	20.10
750.50	6.71	0.48	20.30
788.49	6.89	0.49	20.60
878.67	7.24	0.54	20.75
918.10	8.48	0.63	20.95

**Table 5:** Regression Data.

Solar Irradiance (G)	Short-Circuit Current (I_SC_)	G^2^	I_SC_ × G
449.58	0.27	202,122.20	160.03
516.22	0.31	266,483.10	207.80
593.70	0.35	352,479.70	225.19
608.63	0.37	370,430.50	257.98
661.49	0.39	437,569.00	386.36
719.60	0.44	517,824.20	474.48
750.00	0.48	548,147.70	325.76
788.49	0.49	621,716.50	121.39
878.67	0.54	772,061.00	345.41
918.10	0.63	842,907.60	578.40
**∑G = 6884.48**	**∑I_SC_ = 4.27**	**∑G^2^ = 493,1742**	**∑I_SC_ × G = 3082.8**

**Table 6:** Output parameter of the developed regression model.

Sl. No	Parameter	Values
1	Coefficient of determination	0.96124
2	Standard error	0.0230
4	Standard deviation of residual	0.0217

**Table 7:** Residuals of the model.

Observation	Observed Value	Predicted Value	Residuals (Observed Value-Predicted Value)	Standard Residuals (Residual/Standard Deviationof the Residual)
1	0.27	0.256448069	0.013551931	0.623200484
2	0.31	0.304221679	0.005778321	0.265722458
3	0.35	0.359766386	−0.009766386	−0.449118053
4	0.37	0.370469568	−0.000469568	−0.021593587
5	0.39	0.408364421	−0.018364421	−0.84450815
6	0.44	0.464912776	−0.024912776	−1.145641458
7	0.48	0.450022951	0.029977049	1.378527641
8	0.49	0.499409566	−0.009409566	−0.432709265
9	0.54	0.564058788	−0.024058788	−1.106369887
10	0.63	0.592325796	0.037674204	1.732489817

**Table 8:** Reduction in short-circuit current due to deposition of different size of dust particles.

Dust Particle Size (micron)	Short-Circuit Current (amp)	Reduction in Short-Circuit Current (%)
T_0_	0.50	NA
T_1_	0.43	15.68
T_2_	0.37	27.45
T_3_	0.31	39.21
T_4_	0.28	45.09
T_5_	0.26	49.01

**Table 9:** Dust pollutant size impact on solar Irradiance under the same deposition density.

Pollutant Size (µ)	Simulated Solar Irradiance G_S_ (W/m^2^)	Reduction in Solar Irradiance RSI (%)
600–850 µ	702.85	12.14
300–600 µ	617.14	22.85
150–300 µ	531.43	33.57
75–150 µ	488.57	38.92
Less than 75 µ	460.00	42.50

## Data Availability

Not applicable.

## Sample Availability

Samples of the compounds are available from the authors.
